# Drug therapy for alcohol dependence in primary care in the UK: A Clinical Practice Research Datalink study

**DOI:** 10.1371/journal.pone.0173272

**Published:** 2017-03-20

**Authors:** Andrew Thompson, Darren M. Ashcroft, Lynn Owens, Tjeerd P. van Staa, Munir Pirmohamed

**Affiliations:** 1 Wolfson Centre for Personalised Medicine, Institute of Translational Medicine, University of Liverpool, Liverpool, United Kingdom; 2 Centre for Pharmacoepidemiology and Drug Safety, University of Manchester, Manchester Academic Health Sciences Centre (MAHSC), Manchester, United Kingdom; 3 Hepatology, Royal Liverpool University Hospital Trust, Ward 5z Link, Prescot Street, Liverpool, United Kingdom; 4 Health eResearch Centre, Farr Institute, University of Manchester, Manchester, United Kingdom; 5 Division of Pharmacoepidemiology & Clinical Pharmacology, Utrecht University, Utrecht, The Netherlands; Harvard Medical School, UNITED STATES

## Abstract

**Aim:**

To evaluate drug therapy for alcohol dependence in the 12 months after first diagnosis in UK primary care.

**Design:**

Open cohort study.

**Setting:**

General practices contributing data to the UK Clinical Practice Research Database.

**Participants:**

39,980 people with an incident diagnosis of alcohol dependence aged 16 years or older between 1 January 1990 and 31 December 2013.

**Main outcome measure:**

Use of pharmacotherapy (acamprosate, disulfiram, naltrexone, baclofen and topiramate) to promote abstinence from alcohol or reduce drinking to safe levels in the first 12 months after a recorded diagnosis of alcohol dependence.

**Findings:**

Only 4,677 (11.7%) of the cohort received relevant pharmacotherapy in the 12 months following diagnosis. Of the 35,303 that did not receive pharmacotherapy, 3,255 (9.2%) received psychosocial support. The remaining 32,048 (80.2%) did not receive either mode of treatment in the first 12 months. Factors that independently reduced the likelihood of receiving pharmacotherapy included: being male (Odds Ratio [OR] 0.74; 95% CI 0.69 to 0.78); older (65-74 years: OR 0.61; 95% CI 0.49 to 0.77); being from a practice based in the most deprived quintile (OR 0.58; 95% CI 0.53 to 0.64); and being located in Northern Ireland (OR 0.78; 95% CI 0.67 to 0.91). The median duration to initiation of pharmacotherapy was 0.80 months (95% CI 0.70 to 1.00) for acamprosate and 0.60 months (95% CI 0.43 to 0.73) for disulfiram. Persistence analysis for those receiving acamprosate and disulfiram revealed that many patients never received a repeat prescription; persistence at 6 months was 27.7% for acomprosate and 33.2% for disulfiram. The median duration of therapy was 2.10 months (95% CI 1.87 to 2.53) for acamprosate and 3.13 months (95% CI 2.77 to 3.36) for disulfiram.

**Conclusion:**

Drug therapy to promote abstinence in alcohol dependent patients was low, with the majority of patients receiving no therapy, either psychological or pharmacological. When drug therapy was prescribed, persistence was low with most patients receiving only one prescription. Our data show that treatment for alcohol dependence is haphazard, and there is an urgent need to explore strategies for improving clinical management of this patient group.

## Introduction

The term alcohol use disorder (AUD) spans a spectrum of conditions, with alcohol dependence considered the most extreme phenotype. Alcohol dependence manifests from chronic, repeated exposure to ethanol which results in a cluster of behavioural, neurological, and physiological adaptations [1]. Current therapeutic options for those with alcohol dependence are limited, and most studies examining outcomes of individuals attending for treatment find that 70–80% will relapse in the first year, with the highest rate of relapse taking place in the first 3 months [2, 3]. Those that remain abstinent from alcohol for the first year after treatment have a relatively low risk of relapse thereafter [4].

In the UK, the National Institute for Health and Care Excellence (NICE) provide treatment guidelines that advocate psychological/social support and pharmacotherapy [5]. Psychological interventions represent an important therapy in this patient group, but often need to be used in conjunction with other forms of treatment, including pharmacotherapies. The guidelines state that after a successful withdrawal for people with moderate and severe alcohol dependence, acamprosate, oral naltrexone or disulfiram should be considered in combination with a psychological intervention. In the UK, acamprosate, naltrexone and disulfiram have been the traditional medications indicated for promoting abstinence and for reducing alcohol consumption, with nalmefene being approved by NICE in 2014. Acamprosate was first authorised for use in the UK in 1995, naltrexone in 2011, and disulfiram in 1994, but naltrexone and disulfiram were used off-label before these dates. Each has its own specific mechanism of action [6] but none are universally accepted by healthcare professionals, and pharmacotherapy appears to be under-utilised in this population.

A US survey revealed that only 9% of people needing treatment for alcohol dependence received pharmacotherapy for the disorder [7]. In England, data from the Health and Social Care Information Centre indicate that there has been a 53% rise in the prescription of acamprosate and disulfiram between 2003 and 2014 [8]; in 2014 there were a total of 193,216 prescriptions in primary and secondary care. However, this data only quantifies the absolute number of prescriptions, not the number of patients receiving these drugs or any further details such as patient demographics. Little is known about prescribing practices for alcohol dependence in primary care. By exploring the extent to which medications are utilised in specific healthcare settings, we can help provide a baseline for future quality targets and perhaps motivate healthcare providers to consider their current practice against quality standards provided in national guidelines.

Here we used the Clinical Practice Research Datalink (CPRD) to explore treatment utilisation in primary care patients with alcohol dependence, and investigate factors that may influence the choice of treatment pathway. Specifically, we examined temporal trends in prescribing, and factors associated with initiation and persistence with drug treatment for alcohol dependence.

## Materials and methods

The data was sourced from the Clinical Practice Research Datalink, a large electronic health record database which contains anonymised primary care data from about 8% of the UK population. CPRD has been shown to be broadly representative of the UK population in terms of age, gender and ethnicity [9] and at the time of data analysis covered 689 contributing general practices with approximately 14 million patients. Data in the CPRD is routinely collected and includes patient demographic information, diagnoses, hospital referrals, prescription details, laboratory test results, and lifestyle variables such as smoking status and body mass index. Detailed information on CPRD, including its quality control procedures, is available elsewhere [9, 10]. The study was approved by the Independent Scientific Advisory Committee on 27^th^ August 2014 (protocol 14_151). Patient and practice confidentiality was maintained in accordance with the CPRD policy on personal data.

Diagnostic information is recorded in CPRD by General Practitioners using a hierarchical system of coding known as Read codes [11]. Due to the large number of coding options available for many diagnoses we undertook a systematic, multistep process to produce a case definition for alcohol dependence when using CPRD:

1. Exploration of the Read code database identified an initial list of 289 codes of interest, of which 144 were excluded after review because of lack of relevance or potential to indicate alcohol dependence;
2. Following extraction of the remaining codes in CPRD, we undertook several queries relating to code frequency. We also explored patients that only had a single record of the Read codes under investigation. This exercise resulted in the decision to remove all codes relating to screening tools (e.g. Fast Alcohol Screening Test and AUDIT), as these appeared many times as the only relevant record, and those codes appearing ≤10 occasions in the entire cohort;
3. All codes which describe an alcohol consumption pattern rather than an overt diagnosis were excluded because ‘hard’ clinical diagnoses are more readily recorded in CPRD;
4. Our initial code list included a number of codes that referred to some level of treatment for alcohol misuse. These codes vary from brief interventions to admission to a detoxification facility. It was decided that these codes should only be used in conjunction with other hard codes for alcohol dependence in order to examine treatment patterns rather than for identification as a case, unless clinical consensus deemed otherwise; and
5. Two clinical experts in alcohol/addiction were asked to independently review the remaining 84 codes and dichotomise according to whether they believed the codes likely identified a case of alcohol dependence. Where agreement was clear between reviewers, codes were included or excluded accordingly. Discordant codes were discussed between the reviewers and members of the research team, and grouped according to consensus. This process resulted in 47 codes being included in the case definition for “alcohol dependence”. The clinical code list is available from www.clinicalcodes.org [12] and S1 Table.

We utilised an 'open' cohort study design, such that each patient's time at risk commenced at a different time point, and some exited prior to the end of the study period. The study population consisted of all individuals in CPRD aged 16 years or older between 1 January 1990 and 31 December 2013. An incident case (i.e., first occasion of a Read code included in case definition) of alcohol dependence was defined as: patients had to be registered at the start of the year (1 January), be in their current registration phase with the practice, be registered for at least 1 year, and have no recorded history of alcohol dependence prior to start of the year. The date of incident diagnosis for alcohol dependence is known as the index date.

All patients were followed-up for treatment outcomes from the index date for either 12 months, the date of transfer of the patient out of the practice, the patient’s death as recorded in the CPRD database, or end of study period (31 December 2014), whichever came first. The main treatment outcome was whether the patient was or was not treated with medication to promote abstinence from alcohol or reduce drinking to safe levels. The medications selected were acamprosate, disulfiram, naltrexone, baclofen and topiramate; due to off-label use and comparatively few relevant prescriptions, baclofen and topiramate are clustered as ‘other’ for the purposes of data analysis. Nalmefene was provisionally considered but excluded due to the low number of patients in the cohort that received the drug. Some selected medications have alternative indications for use, such as baclofen for spasticity and topiramate for epilepsy. For each medication the British National Formulary (version 66) was consulted to identify other indications. Medications were deemed prescribed for other conditions if a patient had a diagnosis that was relevant to other indications up to 18 months before the medication in question being prescribed.

Secondary outcomes included whether patients were referred for adjunct psychosocial support, defined by referral codes (available at clinicalcodes.rss.mhs.man.ac.uk [12]), and the factors associated with patients receiving a relevant pharmacotherapy. These factors were; gender; age across seven bands (16–24, 25–34, 35–44, 45–54, 55–64, 65–74, and 75 years and above);; UK Home Nations (England, Northern Ireland, Scotland, and Wales); year of diagnosis across 5 bands (1990–1994, 1995–1999, 2000–2004, 2005–2009, and 2010–2013); and practice-level Index of Multiple Deprivation (IMD) 2010 quintiles (1 = least deprived; 5 = most deprived). For IMD, the general practice postcode is linked via lower layer super output areas or datazone in Scotland, and nation-specific IMD scores extracted. These scores are not directly comparable but act as a proxy with broadly similar measures across seven domains: 1) income, 2) employment, 3) health deprivation and disability, 4) education, skills, and training, 5) barriers to housing and services, 6) crime, and 7) living environment. Univariable and multivariable logistic regression was used to analyse factors associated with prescribing relevant medication. In the multivariable logistic model, all risk factors were included.

Where medication was prescribed in the first 12 months after the index date, a subgroup analysis for initiation of treatment and prescribing persistence were performed in patients diagnosed between 2008 and 2013. These analyses were only performed for acamprosate and disulfiram because of the low levels of utilisation of other pharmacotherapies. Initiation is described as the time from diagnosis to first prescription. Prescribing persistence is defined as a patient having a record of a repeat prescription within 90 days of the expected end date of their last prescription. For example, if a patient had an acamprosate prescription that lasts 28 days, a repeat would need to be issued within 90 days otherwise persistence would be deemed to have “failed” and the patient would be censored. This timeframe was selected based on consensus and similar analysis using CPRD [13]. Patients were followed for persistence for 18 months after the issue of the first prescription. This timeframe allows observation of persistence beyond the initial six months of pharmacotherapy recommended by NICE, and particularly the use of acamprosate beyond its 12 month UK license where continued use requires written consent from the patient. Patients were only able to contribute one event unless both acamprosate and disulfiram commenced on the same date, which resulted in both medications being considered. Patients who died or left the practice during the time period for repeat prescribing were censored. Kaplan-Meier survival analysis was used to estimate the duration of medication initiation and persistence. Hazard ratios were calculated from a Cox proportional hazard model for both initiation and persistence analysis, which was adjusted for age at diagnosis, gender, and IMD quintile. Hazard ratios were further adjusted in the persistence analysis model for medication initiation time (i.e., days from diagnosis to commencing medication).

All analyses were conducted using R [14]. Statistical significance was considered as *P* < 0.05. Strengthening the Reporting of Observational Studies in Epidemiology (STROBE) guidelines were utilised, where applicable (S2 Table).

## Results

When our selection criteria were applied, we identified 39,980 eligible patients; 26,994 (67.5%) were males, and the mean age at diagnosis was 45 years (SD = 14) (Table 1).

**Table 1:** Baseline characteristics of cohort.

Characteristic	Patients (*N*)
**Sex**	
Male	26994 (67.5%)
Female	12986 (32.5%)
**Age(mean±SD)**	45±14
**Age Category**	
16–24	2324 (5.8%)
25–34	6841 (17.1%)
35–44	10688 (26.7%)
45–54	9998 (25.0%)
55–64	6333 (15.8%)
65–74	2667 (6.7%)
≥75	1129 (2.8%)
**IMD quintile**	
1 (low)	5659 (14.2%)
2	6753 (16.9%)
3	6830 (17.1%)
4	8955 (22.4%)
5 (high)	11783 (29.5%)
**UK Home Nation**	
England	28429 (71.1%)
Northern Ireland	2177 (5.4%)
Scotland	6175 (15.4%)
Wales	3199 (8.0%)

Of this cohort, only 4,677 (11.7%) were treated with a relevant pharmacotherapy in the 12 months following incident diagnosis. Eight-hundred and one (17.1%) of the patients who received medication also received adjunct psychosocial support during the same time period. Of the 35,303 that did not receive pharmacotherapy, 3,255 (9.2%) were reported to have received psychosocial support. The remaining 32,048 (80.2%) did not receive either mode of treatment in the first 12 months after diagnosis. Of the cohort, 167 (0.004%) patients were excluded from being considered as treated with pharmacotherapy because of other potential indications for the medication.

Prescribing practices have changed over time with the wider availability of different treatments. Table 2 shows the number of medication users per drug by calendar year. All prescriptions were considered, and thus 556 patients contributed to more than one medication group in a given year over the study period. During the early 1990s disulfiram was the predominant drug. By the late 1990s acamprosate had been introduced and became the most likely drug to be prescribed. The proportions of each drug prescribed remained relevantly stable from 2000 onwards, when patients were around two to three times more likely to receive acamprosate than disulfiram. Naltrexone and other medications were used infrequently during the entire analysis period. The absolute number of patients receiving psychosocial support (both as a single therapy and as an adjunct to pharmacotherapy) increased until the mid-2000s and then has remained relatively stable.

**Table 2:** Alcohol dependence cohort, number of patients treated with pharmacotherapies and number of patients receiving psychosocial support, by calendar year.

		Total number of alcohol dependent patients treated (%)					
Year of diagnosis	Alcohol dependent population	With acamprosate	With disulfiram	With naltrexone	With other	Pharmacotherapy with adjunct psychosocial treatment	Psychosocial treatment only
1990	208	0 (—)	16 (7.7)	0 (—)	0 (—)	0 (—)	0 (—)
1991	318	0 (—)	16 (5.0)	0 (—)	0 (—)	0 (—)	0 (—)
1992	371	0 (—)	20 (5.4)	0 (—)	0 (—)	0 (—)	1 (0.3)
1993	424	0 (—)	18 (4.2)	0 (—)	2 (0.5)	0 (—)	1 (0.2)
1994	433	0 (—)	20 (4.6)	0 (—)	0 (—)	0 (—)	0 (—)
1995	515	0 (—)	25 (4.9)	0 (—)	1 (0.2)	0 (—)	2 (0.4)
1996	592	5 (0.8)	15 (2.5)	0 (—)	0 (—)	1 (0.2)	8 (1.4)
1997	880	32 (3.6)	27 (3.1)	0 (—)	3 (0.3)	5 (0.6)	15 (1.7)
1998	1,047	45 (4.3)	22 (2.1)	2 (0.2)	1 (0.1)	5 (0.5)	25 (2.4)
1999	1,365	75 (5.5)	43 (3.2)	2 (0.1)	2 (0.1)	6 (0.4)	60 (4.4)
2000	1,781	151 (8.5)	57 (3.2)	4 (0.2)	3 (0.2)	18 (1.0)	42 (2.4)
2001	2,058	170 (8.3)	63 (3.1)	3 (0.1)	2 (0.1)	22 (1.1)	68 (3.3)
2002	2,438	229 (9.4)	87 (3.6)	6 (0.2)	3 (0.1)	31 (1.3)	103 (4.2)
2003	2,651	259 (9.8)	98 (3.7)	1 (<0.1)	3 (0.1)	39 (1.5)	169 (6.4)
2004	2,928	262 (8.9)	115 (3.9)	4 (0.1)	6 (0.2)	45 (1.5)	197 (6.7)
2005	2,901	258 (8.9)	121 (4.2)	4 (0.1)	6 (0.2)	61 (2.1)	311 (10.7)
2006	2,839	291 (10.3)	111 (3.9)	1 (<0.1)	8 (0.3)	80 (2.8)	232 (8.2)
2007	2,617	254 (9.7)	121 (4.6)	2 (0.1)	10 (0.4)	67 (2.6)	260 (9.9)
2008	2,696	275 (10.2)	114 (4.2)	2 (0.1)	11 (0.4)	75 (2.8)	272 (10.1)
2009	2,599	273 (10.5)	115 (4.4)	4 (0.2)	6 (0.2)	80 (3.1)	347 (13.4)
2010	2,289	282 (12.3)	103 (4.5)	6 (0.3)	11 (0.5)	94 (4.1)	315 (13.8)
2011	2,221	267 (12.0)	97 (4.4)	6 (0.3)	7 (0.3)	73 (3.3)	280 (12.6)
2012	1,992	213 (10.7)	59 (3.0)	9 (0.5)	15 (0.8)	52 (2.6)	281 (14.1)
2013	1,817	177 (9.7)	55 (3.0)	7 (0.4)	14 (0.8)	47 (2.6)	266 (14.6)

Several factors that were considered in the univariable logistic regression analysis were predictors of treatment with pharmacotherapy (Table 3). Males were significantly less likely to receive pharmacotherapy for their alcohol dependence (OR 0.73; 95% CI 0.68 to 0.78). Those aged 16–24 were significantly less likely to receive medication than those aged 25–54, but the youngest age group were more likely to receive medication than those aged 65 and above. Compared with the least deprived quintile, those from most the deprived quintile were significantly less likely to receive medication (OR 0.62; 95% CI 0.56 to 0.69). Patients from the other deprivation quintiles were also less likely to receive pharmacotherapy compared with the least deprived group, but statistical significance was only observed in the second least deprived quintile (OR 0.85; 95% CI 0.76 to 0.94). Using England as the reference nation, patients diagnosed in Northern Ireland were significantly less likely to receive pharmacotherapy (OR 0.80; 95% CI 0.98 to 0.93), whereas those diagnosed in Scotland (OR 1.49; 95% CI 1.38 to 1.61 or Wales (OR 1.41; 95% CI 1.27 to 1.57) were significantly more likely. The likelihood of receiving pharmacotherapy appears to have increased over time. Patients diagnosed with alcohol dependence in more recent years (inclusive of 2000–2013) were significantly more likely to receive medication than those diagnosed in earlier years (1990–1994).

**Table 3:** Univariable and Multivariable odds ratios analysis for the association between individual patient factors associated with prescribing drugs for alcohol dependence.

Risk Factor	Univariable	95% CI	*P*	Multivariable*	95% CI	*P*
	OR			OR		
**Gender – Men vs Women**	0.73	0.68 to 0.78	<0.0005	0.74	0.69 to 0.78	<0.0005
**Age (years)**						
16–24	1.00	Reference		1.00	Reference	
25–34	1.80	1.53 to 2.12	<0.0005	1.92	1.63 to 2.27	<0.0005
35–44	2.02	1.73 to 2.37	<0.0005	2.12	1.81 to 2.49	<0.0005
45–54	1.56	1.33 to 1.83	<0.0005	1.60	1.36 to 1.89	<0.0005
55–64	1.09	0.92 to 1.30	0.330	1.10	0.92 to 1.31	0.301
65–74	0.62	0.49 to 0.77	<0.0005	0.61	0.49 to 0.77	<0.0005
≥75	0.21	0.13 to 0.33	<0.0005	0.21	0.13 to 0.32	<0.0005
**IMD**						
1 (low)	1.00	Reference		1.00	Reference	
2	0.85	0.76 to 0.94	0.002	0.83	0.75 to 0.93	<0.001
3	0.94	0.84 to 1.04	0.208	0.91	0.82 to 1.01	0.071
4	0.94	0.85 to 1.04	0.219	0.92	0.83 to 1.02	0.101
5 (high)	0.62	0.56 to 0.69	<0.0005	0.58	0.53 to 0.64	<0.0005
**UK Home Nation**						
England	1.00	Reference		1.00	Reference	
Northern Ireland	0.80	0.68 to 0.93	0.004	0.78	0.67 to 0.91	0.002
Scotland	1.49	1.38 to 1.61	<0.0005	1.59	1.46 to 1.72	<0.0005
Wales	1.41	1.27 to 1.57	<0.0005	1.44	1.29 to 1.60	<0.0005
**Year of Diagnosis**						
1990–1994	1.00	Reference		1.00	Reference	
1995–1999	1.22	0.97 to 1.57	0.097	1.20	0.94 to 1.54	0.141
2000–2004	2.37	1.92 to 2.97	<0.0005	2.29	1.85 to 2.86	<0.0005
2005–2009	2.65	2.15 to 3.32	<0.0005	2.49	2.01 to 3.11	<0.0005
2010–2013	2.98	2.41 to 3.74	<0.0005	2.81	2.26 to 3.52	<0.0005

OR = odds ratio, CI = confidence interval

*Each risk factor is independently adjusted for other risk factors.

In the multivariable analysis, several factors determined the likelihood of receiving a prescription of a drug for alcohol dependence (Table 3). Males were less likely to receive drug therapy (Odds Ratio 0.74; 95% CI 0.69 to 0.78). There was a decreased likelihood of receiving a drug if the patient was from a practice located in the most deprived quintile (Odds Ratio 0.58; 95% CI 0.53 to 0.64); if they were diagnosed at an older age (age 65–74 years Odds Ratio 0.61; 95% CI 0.49 to 0.77; age 75 years and above Odds Ratio 0.21; 95% CI 0.13 to 0.32); and if the patient resided in Northern Ireland (Odds Ratio 0.78; 95% CI 0.67 to 0.91).

There was increased likelihood of receiving a prescription if the patient was diagnosed more recently (incident diagnosis made 2010–2013 Odds Ratio 2.81; 95% CI 2.26 to 3.52); diagnosed in Scotland (Odds Ratio 1.59; 95% CI 1.46 to 1.72) or Wales (Odds Ratio 1.44; 95% CI 1.29 to 1.60); and received a diagnosis at certain ages (age 25–34 years Odds Ratio 1.92; 95% CI 1.63 to 2.27; age 35–44 years Odds Ratio 2.12; 95% CI 1.81 to 2.49; age 45–54 years Odds Ratio 1.60; 95% CI 1.36 to 1.89).

**Fig 1:**
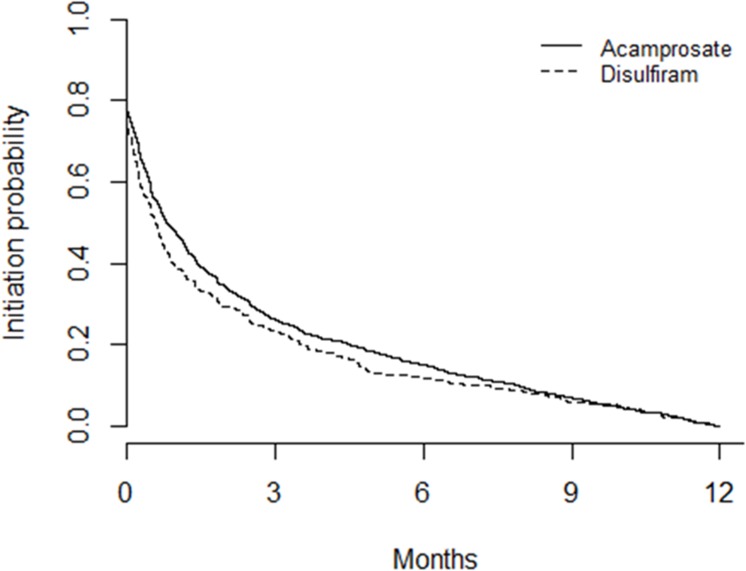
Initiation of prescribing for acamprosate and disulfiram. Number at start of analysis: acamprosate = 1,257; disulfiram = 394.

Fig 1 illustrates the association between time since date of diagnosis for alcohol dependence and initiation of pharmacotherapy. The median duration to initiation of pharmacotherapy was 0.80 months (95% CI 0.70 to 1.00) for acamprosate and 0.60 months (95% CI 0.43 to 0.73) for disulfiram. By 6 months in those patients who received pharmacotherapy, 85% of patients had been commenced on acamprosate and 88% have been commenced on disulfiram within their respective subgroups. Males were more likely to be commenced on medication later than females (OR 1.20; 95% CI 1.09 to 1.33); neither of the variables explored (i.e., gender and IMD) were statistically significant for predicting medication initiation.

**Fig 2:**
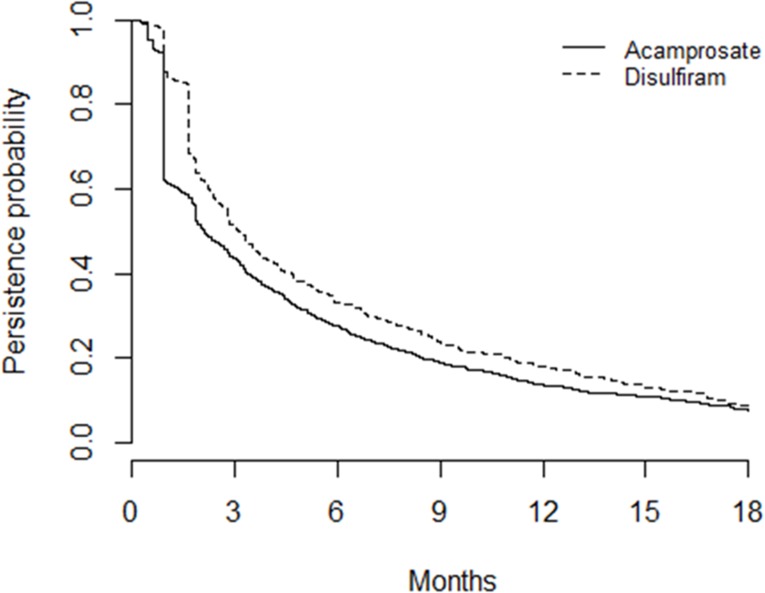
Persistence of prescribing for acamprosate and disulfiram for 18 months after the date of first prescription. Number at start of analysis: acamprosate = 1,257; disulfiram = 394.

Persistence analysis revealed that many patients never received a repeat prescription, meaning there was a large reduction in the number of persistent patients within two months of the first prescription being issued (Fig 2). Over 6 months, persistence was 27.7% for acamprosate and 33.2% for disulfiram; over 12 months persistence was 13.7% and 17.9%, respectively; and over 18 months persistence was 7.5% and 8.8%, respectively. The median duration of acamprosate therapy was 2.10 months days (95% CI 1.87 to 2.53), whereas the median duration for disulfiram was 3.13 months (95% CI 2.77 to 3.36). Calculated hazard ratios suggest that younger patients were likely to stop treatment earlier (HR_adj_ 0.99; 95% CI 0.98 to 0.99), and those from the most deprived quintile, compared with the least deprived, were also more likely not to persist with treatment (HR_adj_ 0.74; 95% CI 0.63 to 0.88); there was no significant effect for gender or time between being diagnosed and treatment being initiated.

## Discussion

Evidence [15] and guidelines [5] suggest that pharmacotherapy should be considered as a treatment option for patients with alcohol dependence. Our findings however highlight potentially missed opportunities to engage patients on appropriate treatment pathways in UK primary care. In our cohort of almost 40,000, only 11.7% received pharmacotherapy within 12 months of being diagnosed. Treatment persistence analysis revealed that many of those that were prescribed acamprosate or disulfiram did not receive a second prescription within 90 days of the expected end of their first prescription, potentially highlighting a time point in the treatment pathway where extra attention is required to maintain engagement. Treatment retention and adherence are well-established predictors of long term outcomes [16], and it has been reported that patients who receive an alcohol-related medication have lower healthcare utilisation than those who do not [17, 18].

Although the utilisation of pharmacotherapy was limited, certain trends deserve discussion and potential further investigation. First, females were significantly more likely to receive pharmacotherapy. It is known that females are more likely to suffer harm from alcohol [19, 20] but this does not explain the reported gender-gap. It is posited that females may be more willing to engage with treatment pathways and thus accept intervention for their alcohol dependence [21]. Conversely, reports suggest that females encounter a number of barriers when trying to access substance abuse services [22], meaning their condition might be more severe when they present and pharmacotherapy is more likely to be considered.

Second, although no linear trend was observed for deprivation and likelihood of receiving pharmacotherapy, those from the most deprived quintile were least likely to receive medication. Similar findings have been reported in Australia, where those in the most socially disadvantaged areas were 4.5 and 5.2 times less likely to receive naltrexone and acamprosate, respectively, compared with the least disadvantaged [23]. Given the increased harm from alcohol reported in this group (i.e. the alcohol harm paradox) [24] it is perhaps surprising that they appear to be poorly managed against quality standards [5]. Indeed, this apparent health inequality could be a reason for poor health outcomes in those living in the most deprived areas.

Third, the upward trend in prescribing in recent years can be seen as encouraging but appropriate training and support needs to be given to GPs and other primary care staff to ensure they have knowledge, skills and confidence that enables them to deliver appropriate clinical pathways, including both psychosocial support and pharmacotherapy. General practitioners are recognised as being well placed to identify [25] and treat [26] patients with alcohol-use disorder, although the principal care of these patients is often devolved to secondary or tertiary services. It should be noted however that other factors may determine pharmacotherapy use for alcohol dependence including comorbid conditions and drug interactions, patients’ preferences and cognitive function, and evidence suggesting limited efficacy [27]. Furthermore, as advances are made in certain areas (e.g. pharmacogenomics) other factors may need to be considered to ensure good practice.

Data from the United States (US) [7] generally support our findings for the UK. However, it should be noted that the definition of alcohol dependence between studies may vary and comparisons should be considered with this caveat. Furthermore, the figures presented within our manuscript are limited to primary care and individuals within the CPRD system. It was estimated that 9% of the US population with alcohol dependence received at least one pharmacotherapy, compared with 11.7% in the present study. In the US there was an increase in the number of prescriptions for alcohol dependence, with an annual growth rate of 12.9% between 2003 and 2007. Furthermore, acamprosate was soon established as the market leader and the number disulfiram prescriptions reduced.

The use of standardised methods across three healthcare databases in the US revealed some variation in the level of prescribing in alcohol use disorder treatment. The number of patients that filled at least one prescription for alcohol pharmacotherapy in 2006 ranged from 2.6 to 16.4% [28]. Results from a study investigating medication use in the biggest of the aforementioned databases, the Veterans Health Administration, found that approximately 3% of militarily veterans received pharmacotherapy for an alcohol use disorder [29]. Population-wide data from Australia also suggest that a low number of patients with alcohol dependence are treated with pharmacotherapy, estimated to be between 2.7–3% [30]. Follow-up surveys with healthcare providers suggested several prominent barriers to prescribing including, perceived low patient demand, pharmacy procedures or formulary restrictions, lack of provider skills or knowledge regarding pharmacotherapy for alcohol dependence, and lack of confidence in treatment effectiveness [31]. Some of these barriers were drug-specific meaning that individual strategies may be required to improve prescribing levels of each drug.

Persistence at 6 months for the two medications analysed in our study was 25.6% for acamprosate and 31.3% for disulfiram. Guidelines from NICE [5] generally recommend prescribing these medications for up to 6 months initially, or for longer if the drug is effective and/or the patient wishes to continue. A study investigating persistence for naltrexone reported 14.2% of patients remained engaged at 6 months [32]. Furthermore, other data suggest that only 25% and 15% of patients prescribed naltrexone and acamprosate, respectively, received at least 3 months of treatment [30]. Although we did not analyse naltrexone because of the low number of prescriptions, these comparisons highlight the global issue of treatment retention in this clinical cohort. This is salient given the reported improved outcomes in patients that remain engaged with treatment for at least one year [4]. The rate of pharmacotherapy initiation in those that received acamprosate or disulfiram was highest in the first month after recorded diagnosis; although this had little effect on treatment persistence.

Other psychiatric conditions are generally managed to a greater extent with pharmacotherapy. Further data from the Veterans Affairs healthcare system demonstrate that patients with dual diagnosis (i.e. alcohol use disorder and at least one other comorbid psychiatric condition) were less likely to receive pharmacotherapy for their alcohol disorder than their co-occurring condition [33]. The proportion receiving pharmacotherapy for alcohol ranged from 7% to 11%, whereas psychiatric conditions (i.e. bipolar, schizophrenia, major depressive disorder and posttraumatic stress disorder) ranged from 69 to 82%. Tobacco addiction pharmacotherapy was also investigated with receipt reported at 34%. On balance it would therefore seem reasonable to conclude that pharmacotherapy is underutilised in the treatment of alcohol dependence.

Psychosocial interventions encompass a number of techniques that are widely used in healthcare systems and non-statutory services. Some interventions lack strong evidence but many are considered to be superior to treatment as usual or control conditions [34]. Our findings suggest that approximately four in five of those that received medication were not given adjunct psychosocial support, despite NICE guidelines recommending that pharmacotherapy should be prescribed in combination with a psychological intervention. Current guidelines recommend that pharmacotherapy should be delivered with psychosocial social support, offering a further area to consider in improving care quality standards in this population [5].

This study has several strengths and limitations above those normally cited in database studies. First, CPRD is a database that reflects primary care. Theoretical and empirical evidence suggests that many patients who are dependent on alcohol may attend secondary or tertiary services to receive either planned or emergency care that is related to their alcohol consumption. Although not unique, this treatment pattern means that patients on these pathways require GPs to follow-up and subsequently record any diagnosis in their primary care records to be eligible for our cohort. Furthermore, many individuals with alcohol dependence do not seek treatment, which might mean that our sample does not represent the entire population figure. Second, medication use was defined as a prescription in the GP system, and not by actual issue of medication to the patient. Therefore, misclassification of medication use is possible since prescriptions recorded in the GP system may not have been dispensed by the pharmacy, or actually used by the patient. Furthermore, we are unable to monitor actual patient engagement with pharmacotherapy, even if a repeat prescription is issued, as this does not guarantee that the medication is being used as directed by the medical practitioner. Third, we are unable to define the treatment goals (abstinence vs. harm reduction) of the cohort under investigation. The variation in treatment goals might account for some of the variation in the type of pharmacotherapy prescribed. For example, those targeting harm reduction via ‘controlled’ alcohol consumption would not be prescribed disulfiram. Finally, data for deprivation status was obtained at practice level rather than for each individual. Despite these limitations, we believe that the process undertaken to achieve our case definition is a particular strength and increases the likelihood of having a population that is representative of those with alcohol dependence. Furthermore, our sample is taken from the general population rather than more homogeneous samples that have been used in previous studies, such as the Veterans, meaning our findings are more generalisable.

In conclusion, pharmacotherapy in this cohort has the potential to increase success of clinical pathways but appears to be grossly underutilised in practice. This patient cohort is often difficult to engage and resistant to change. This may be compounded however by lack of education and poor understanding of what therapies will be effective for each individual. This lack of stratification early in the treatment pathway may lead to a negative treatment outcome that disengages the patient, but more research is required to explore moderators that have biological plausibility and clinical utility. Finding effective strategies to promote abstinence in patients with alcohol use disorder should remain a priority given the associated positive effects. Further measures to improve adherence to national guidelines are needed to improve the overall clinical management of patients with alcohol dependence.

## Supporting information

S1 Table Read Codes included in the case definition for “alcohol dependence”. (DOCX)

S2 Table STROBE statement checklist. (DOC)
