# Supplementary material for: Drug therapy for alcohol dependence in primary care in the UK: A Clinical Practice Research Datalink study
Source: PLoS One. 2017 Mar 20;12(3):e0173272. doi: 10.1371/journal.pone.0173272 (PMC5358741; doi:10.1371/journal.pone.0173272)
Supplement: S1 Table — (DOCX) [file pone.0173272.s001.docx]

**Supplemental Material 1**

**S1 Table: Read Codes included in the case definition for “alcohol dependence”.**

| **Read Code** | **Description** | **Frequency of incident cases (%)** |
| --- | --- | --- |
| Eu10211 | [X]Alcohol addiction | 0.63 |
| Eu10800 | [X]Alcohol withdrawal-induced seizure | 0.79 |
| Eu10712 | [X]Chronic alcoholic brain syndrome | 0.04 |
| Eu10212 | [X]Chronic alcoholism | 0.43 |
| Eu10411 | [X]Delirium tremens, alcohol induced | 0.34 |
| Eu10213 | [X]Dipsomania | 0.00 |
| Eu10200 | [X]Mental and behavioural disorders due to use of alcohol: dependence syndrome | 0.26 |
| Eu10300 | [X]Mental and behavioural disorders due to use of alcohol: withdrawal state | 0.03 |
| Eu10400 | [X]Mental and behavioural disorders due to use of alcohol: withdrawal state with delirium | 0.02 |
| E230.00 | Acute alcoholic intoxication in alcoholism | 1.63 |
| E230z00 | Acute alcoholic intoxication in alcoholism NOS | 0.07 |
| E230300 | Acute alcoholic intoxication in remission, in alcoholism | 0.02 |
| E230000 | Acute alcoholic intoxication, unspecified, in alcoholism | 0.04 |
| 8H35.00 | admitted to alcohol detoxification centre | 0.16 |
| E23..00 | Alcohol dependence syndrome | 52.51 |
| E23z.00 | Alcohol dependence syndrome NOS | 1.23 |
| E230.11 | Alcohol dependence with acute alcoholic intoxication | 0.03 |
| Z191.00 | alcohol detoxification | 10.79 |
| 8BA8.00 | alcohol detoxification | 0.05 |
| E010.00 | Alcohol withdrawal delirium | 0.21 |
| E013.00 | Alcohol withdrawal hallucinosis | 0.07 |
| E01y000 | Alcohol withdrawal syndrome | 8.02 |
| G555.00 | Alcoholic cardiomyopathy | 0.52 |
| J612.00 | Alcoholic cirrhosis of liver | 4.25 |
| F11x011 | Alcoholic encephalopathy | 0.15 |
| F394100 | Alcoholic myopathy | 0.06 |
| F375.00 | Alcoholic polyneuropathy | 0.33 |
| J671000 | Alcohol-induced chronic pancreatitis | 0.26 |
| E23..11 | Alcoholism | 13.74 |
| F11x000 | Cerebral degeneration due to alcoholism | 0.01 |
| E012000 | Chronic alcoholic brain syndrome | 0.01 |
| J617000 | Chronic alcoholic hepatitis | 0.05 |
| E231.00 | Chronic alcoholism | 0.94 |
| E231300 | Chronic alcoholism in remission | 0.02 |
| E231z00 | Chronic alcoholism NOS | 0.65 |
| E230100 | Continuous acute alcoholic intoxication in alcoholism | 0.00 |
| E231100 | Continuous chronic alcoholism | 0.09 |
| E010.12 | Delirium tremens | 0.43 |
| E231.11 | Dipsomania | 0.02 |
| E010.11 | DTs – Delirium tremens | 0.22 |
| E230200 | Episodic acute alcoholic intoxication in alcoholism | 0.03 |
| E231200 | Episodic chronic alcoholism | 0.11 |
| E011000 | Korsakov's alcoholic psychosis | 0.28 |
| E011100 | Korsakov's alcoholic psychosis with peripheral neuritis | 0.04 |
| G852300 | Oesophageal varices in alcoholic cirrhosis of the liver | 0.18 |
| E231000 | Unspecified chronic alcoholism | 0.10 |
| C253.00 | Wernicke’s encephalopathy | 0.18 |
